# Mapping Local Cytosolic Enzymatic Activity in Human Esophageal Mucosa with Porous Silicon Nanoneedles

**DOI:** 10.1002/adma.201501304

**Published:** 2015-07-21

**Authors:** Ciro Chiappini, Paola Campagnolo, Carina S. Almeida, Nima Abbassi‐Ghadi, Lesley W. Chow, George B. Hanna, Molly M. Stevens

**Affiliations:** ^1^ Department of MaterialsDepartment of BioengineeringInstitute of Biomedical EngineeringImperial College LondonSW7 2AZLondonUK; ^2^ Department of Surgery and CancerImperial College LondonW2 1PGLondonUK

**Keywords:** diagnostics, intracellular sensing, nanomedicine, nanoneedles, porous silicon

## Abstract

**Porous silicon nanoneedles** can map Cathepsin B activity across normal and tumor human esophageal mucosa. Assembling a peptide‐based Cathepsin B cleavable sensor over a large array of nano­needles allows the discrimination of cancer cells from healthy ones in mixed culture. The same sensor applied to tissue can map Cathepsin B activity with high resolution across the tumor margin area of esophageal adenocarcinoma.

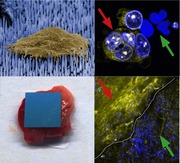

Nanoneedles are developing into versatile nanoscale tools for cell biology and medical intervention.^1^ Their fabrication draws from the experience of vertically aligned nanowires, allowing for the development of solid,^2^ hollow or porous^3^, ^4^ structures from silicon,^5^ carbon,^6^ and several other semiconductors.^7^ Nanoneedles are one of the most sophisticated and minimally invasive tools that allow direct access and manipulation of the intracellular environment. They can deliver a vast range of bioactive molecules and nanoparticles to the cytosol,^2^, ^3^, ^8^ as well as probe the electrical and the biochemical environment inside cells. Metallized silicon nanoneedles can interface in parallel to multiple neuronal cells, enabling either intracellular electrical stimulation or recording of the propagation of action potentials across a synapse.^9^ Arrays of nanoneedle‐based field effect transistors enable the recording of the electrical activity across a network of cardiomyocytes.^5^ Nanoneedles functionalized with proton sensitive fluorescent probes can sense the lowered intracellular pH of cultured cancer cells,^8^ whilst functionalization with caspase sensors enables the identification of apoptosis induced in cells.^10^ Indeed nanoneedles could be a unique tool for large scale mapping of the intracellular environment, inducing minimal perturbation to the target culture or tissue. This capability also enables nanoneedles to compete in the development of medically translatable therapeutic and diagnostic devices that have a low invasive potential.

The recent development of biodegradable porous silicon nanoneedles indicates a path for safe and effective use in vivo through a demonstrated delivery of multiple payloads, including nucleic acids for genetic engineering and neovascularization.^3^, ^8^ The suitability of porous silicon as a material for intracellular sensing stems from its ability to withstand the forces involved in nanoinjection,^3^ its biodegradability,^11^ and elevated biocompatibility as demonstrated by its safe use for intravenous injection, long‐term implants, and brachytherapy.^12^, ^13^, ^14^, ^15^ Porous silicon has a large and spatially constrained interface area for biological interaction,^16^ ideally suited for both electrical and optical biosensing.^17^, ^18^, ^19^

Cathepsin B (CTSB) is a cysteine protease usually confined to the lysosomes and employed as a biomarker in a wide range of solid tumors, correlating with increased invasiveness and poor prognosis.^20^, ^21^ We designed a nanoneedle sensor to detect the cytosolic activity of CTSB, anticipated to be minimal for healthy cells where CTSB is confined to the lysosomes, and increased for cancer cells where CTSB is aberrantly activated in the cytosol.^22^ The sensor consisted of a fluorescently labeled CTSB cleavable peptide covalently conjugated to a nanoneedle array (**Figure** **1**a). Upon interfacing, the nanoneedles in the array interfaced with the intracellular environment and were able to sense intracellular activity. The action of CTSB then cleaved the peptide and released the fluorescent label in the cytosol. It was anticipated that higher CTSB activity would correspond to higher cytosolic fluorescence and allow mapping of CTSB activity within a cell population by fluorescence micro­scopy or flow cytometry.

**Figure 1:**
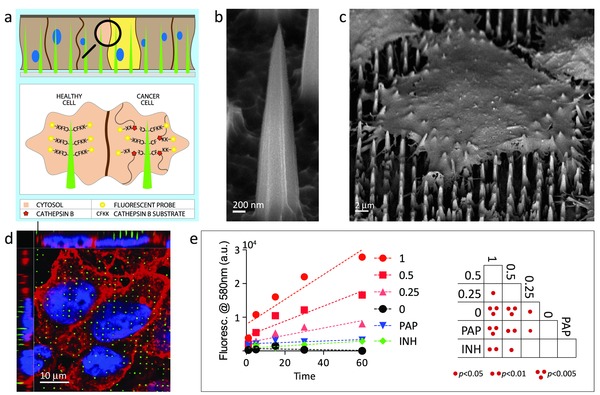
The nanoneedle Cathepsin B sensor. a) Schematic diagram of sensor functionality when interfacing with cells. Nanoneedles interface the cytosol of cells where active CTSB cleaves its CFKK peptide substrate, releasing the linked TAMRA fluorescent probe in the cell cytosol. Higher CTSB activity corresponds to a higher cytosolic fluorescence. Scanning electron microscopy image of b) a nanoneedle and c) a cell seeded over a nanoneedle chip. d) Laser scanning confocal fluorescent microscopy image (LSC) showing nanoneedles interfacing with cells. Nanoneedles in green, cell membrane in red, nucleus in blue. e) Quantification of fluorescence released in solution from nanoneedles exposed to different concentrations of CTSB. Red lines represent CTSB at the indicated concentration in U/mL. PAP represents papain at 1 U mL^–1^, INH represents 1 U mL^–1^ CTSB inhibited with 1 10^–6^ m CA‐074. Negative control is represented by 0. The double entry table reports statistical significance between all pairs.

Here, we present this nanoneedle biosensor that can map the intracellular activity of the cysteine protease CTSB both in cell culture and across a large area of bioptic tissue. The sensor discriminates CTSB positive (+ve) cancer cells from CTSB negative (−ve) cells in a mixed culture. The nanoneedles also sense the difference in CTSB activity in tissue resected from patients with esophageal cancer. The mapping resolution, which approaches the single‐cell level, highlights CTSB +ve and −ve regions within a single tumor resection specimen. These findings highlight a translational potential for nanoneedles as a minimally invasive exploratory tool for a more accurate selection of biopsy sites and for appropriate mapping of target areas during endoscopic mucosal resection.

The manufacturing of nanoneedles is described in our previous study,^3^ and combined photolithography with metal assisted chemical etching to yield approximately 5 μm tall nanoneedles with a 2 μm pitch and a less than 50 nm tip dia­meter (Figure 1b). These nanoneedles interact with the intracellular environment (Figure 1c,d) as we have previously shown.^8^ The CFKK peptide, modified at the terminal lysine with the carboxytetramethylrhodamine (TAMRA) fluorescent probe, acted as a CTSB‐specific sensing element.^23^ The liquid phase deposition of a 3‐aminopropyltriethoxysilane (APTES) layer followed by that of a heterobifunctional amine to sulfhydryl crosslinker covalently conjugated the sensing probe to the porous silicon nanoneedles through the sulfhydryl group on the C‐terminal cysteine. The assembled sensor was able to specifically detect CTSB in solution. A dose‐dependent and time‐dependent response resulted from exposing the sensor to CTSB and measuring the fluorescence released in solution, as expected from an assay for enzymatic activity (Figure 1e). The sensing was specific to CTSB activity as minimal background signal presented: (i) in the absence of CTSB, (ii) when 20 × 10^−6^ m CA‐074 inhibited 1 U mL^−1^ CTSB, or (iii) when the sensor was exposed to 1U mL^−1^ of papain, which belongs to the same cysteine protease family of CTSB.

The nanoneedle sensor allowed for mapping of CTSB activity within cells. We first selected two esophageal epithelial cell lines, HET‐1A (CTSB −ve) and OE33 (CTSB +ve) respectively immortalized and transformed (cancerous) as a matched pair, which displayed a marked difference in their CTSB activity and localization. The cancerous OE33 cells displayed a markedly higher expression of CTSB than HET‐1A cells both at the RNA level (Figure S1, Supporting Information) and protein level (**Figure** **2**a). This higher CTSB expression resulted in an eightfold higher activity for OE33 cultures as shown by a commercial assay performed on cell lysates (Figure 2b). CTSB presented throughout the cytosol of OE33 cells while it was confined inside the endolysosomal system for Het‐1A cells (Figure S2, Supporting Information). The 1.6 × 10^7^ nanoneedles present on each 70 mg chip were interfaced with the cells by centrifugation at 150 rcf, providing a penetration force of 6.4 nN per needle, which lays within the penetration range observed in the literature.^24^, ^25^ When nanoneedles functionalized with the CTSB probe were interfaced with single cultures, they displayed a higher fluorescence originating from OE33 cells compared to Het‐1A cells for all exposure times ranging from 3 to 60 min (Figure 2c,d). The fluorescence intensity was time‐dependent, reaching a maximum at 5 min and then slowly declining for both cell types. To insure the signal observed originated from proteolytic cleavage rather than uptake of adsorbed peptide, we employed the d‐isomer of the peptide as a sensing element, which is impervious to proteolytic cleavage. Both the OE33 and the Het‐1A cells interfaced with the d‐isomer showed minimal cytosolic fluorescence, in comparison to that observed for the l‐isomer (Figure S3, Supporting Information). When employing cytosolic fluorescence as a classifier to distinguish OE33 from Het‐1A cells in isolated cultures, we found a receiver operator characteristic (ROC) with an area under the curve (AUC) of 1 at all times, indicating a correct classification of all OE33 and Het‐1A cells analyzed (Figure S4, Supporting Information).

**Figure 2:**
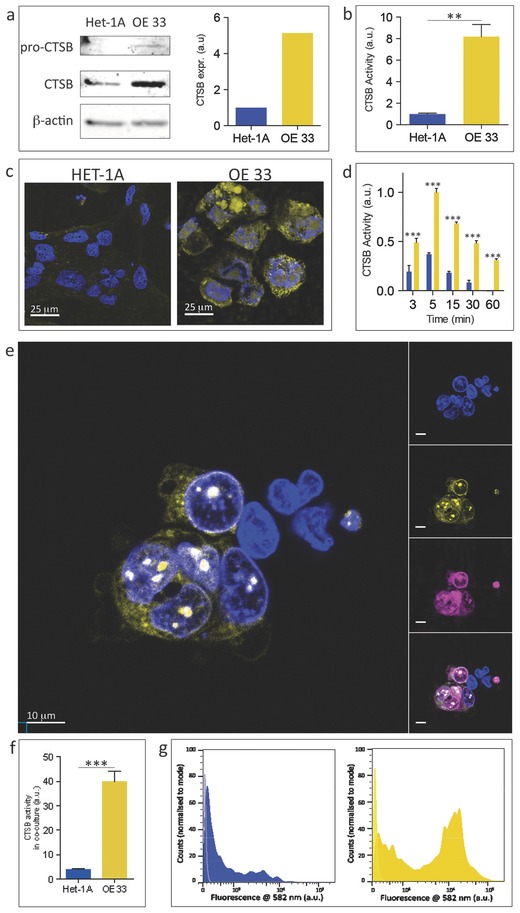
Sensing CTSB activity in cells in culture. a) Sections of western blot membrane cut at the appropriate molecular weight for pro‐CTSB, CTSB and β‐actin showing their expression in HET‐1A and OE33 cells with relative band quantification. Sections are outlined in black. b) Quantification of CTSB activity in HET‐1A and OE33 cells by fluorogenic homogenous assay in cell lysates. c) Representative LSCs of HET‐1A and OE33 cells following application of the nanoneedle sensor for 15 min. Single z‐plane collected through the cytosol above the level of the nanoneedles. Cytosolic fluorescence originates from cleaved CTSB substrate (yellow). Nuclei stained in blue. d) Quantification of the area‐normalized fluorescence cytosolic signal for OE33 (yellow) and HET‐1A (blue) cells interfaced with nanoneedles. The horizontal axis represents interfacing time. e) Representative LSCs of HET‐1A and OE33 cells in coculture following application of the nanoneedles sensor for 15 min. Single z‐plane collected through the cytosol above the level of the nanoneedles. Cytosolic fluorescence originates from cleaved CTSB substrate. Nuclei stained in blue. Insets show separate signal for each fluorescent channel. OE33 cells are stained in magenta (CellVue). f) Quantification of the area‐normalized cytosolic signal for HET‐1A (blue) and OE33 (yellow) cells interfaced with nanoneedles in coculture. g) Intensity histogram for flow cytometric analysis of cytosolic fluorescence for gated HET‐1A (blue) and OE33 (yellow) cells interfaced with nanoneedles in coculture. **p < 0.01, ***p < 0.001.

When we placed the Het‐1A and OE33 cells in coculture and interfaced with the nanoneedle sensor for 15 min, it was still possible to distinguish the higher intracellular fluorescence of CTSB +ve OE33 cells compared to the CTSB −ve Het‐1A cells by confocal microscopy (Figure 2e,f). On average, OE33 cells displayed a cytosolic fluorescence 15‐fold higher than that of Het‐1A cells. In this instance, the AUC for the ROC of the classifier was 0.858 (Figure S4, Supporting Information). Flow cytometry confirmed the fluorescence microscopy observations and provided an unbiased discrimination between OE33 and Het‐1A cells through their cytosolic fluorescence; the AUC was 0.701 (Figure 2g, Figure S4, Supporting Information). The lower AUC observed by flow cytometry likely arose from the inclusion in the analysis of a proportion of cells that did not fully interface with the nanoneedles. Those cells could be easily excluded from the analysis by confocal microscopy as they fully resided above the tips of the nanoneedle array, but could not be discerned by flow cytometry where they contributed to the low fluorescence intensity peak observed for both HET‐1A and OE33 cells (Figure 2g).

We employed the nanoneedle sensor to map CTSB activity within excised tissue samples from esophageal adenocarcinoma patients. An 8 × 8 mm array of nanoneedles could sense CTSB activity within the cytosol of cells on the surface of tissue directly underneath the chip (**Figure** **3**), leaving a clear demarcation line between the area beneath the chip and the one outside (Figure 3a). By comparison, a flat chip was unable to detect any CTSB activity in the same conditions (Figure S5, Supporting Information).

**Figure 3:**
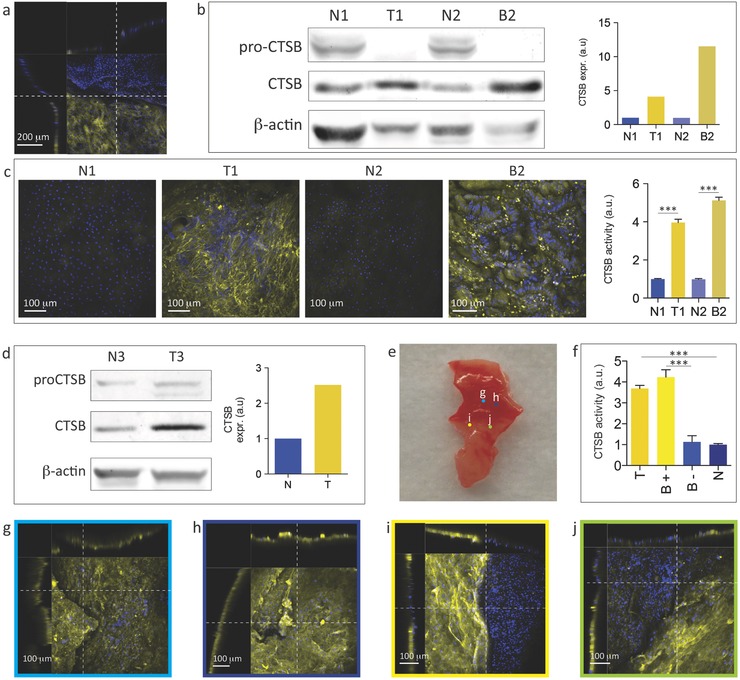
Sensing of CTSB activity in human esophageal tissue. a) LSCs of a human esophageal epithelium sample interfaced with the CTSB nanoneedle sensor for 15 min. The visible demarcation line between fluorescent and nonfluorescent cells originates from the edge of the chip. The side view shows a steep height profile within the tissue at the interfacing edge. b) Sections of western blot membrane cut at the appropriate molecular weight for pro‐CTSB, CTSB and β‐actin showing protein expression with quantification for matching normal (N1 and N2) and diseased (Tumour T1, Barrett's Dysplasia B2) region of tissues from patient 1 (N1, T1) and patient 2 (N2, B2), respectively. Sections are outlined in black. c) LSCs of the esophageal epithelium normal (N1, N2) and diseased (T1, B2) samples interfaced with the CTSB nanoneedle sensor for 15 min. The yellow fluorescence signal originates from cleaved CTSB substrate. Quantification of the fluorescent signal for single cells shows statistically higher fluorescence for diseased samples (T1, B2). d) Sections of western blot membrane cut at the appropriate molecular weight for pro‐CTSB, CTSB and β‐actin showing protein expression with quantification for matching normal (N3) and diseased (T3) region of tissues from patient 3. Sections are outlined in black. e) Photograph of the margin tissue sample from patient 3 immediately prior to interfacing with nanoneedles. Colored dots identified by letters refer to the approximate position of the confocal microscopy images displayed in panels (g–j). f) Quantification of CTSB activity in the different areas of the sample is shown in (e): tumor region (T, panels g, h), CTSB +ve region at the visible tumor margin (B+), CTSB ‐ve region at the visible tumor margin (B‐) (panels i, j), normal region (N, Figure S6). (g, h) LSCs of the areas indicated in (e): (g, h) within the tumor region (i, j) at the tumor margin. The side views show a regular profile without steep changes in height that could affect interfacing or indicate proximity to the edge of the chip. All LSCs XY views are maximum intensity projections along the Z‐axis, and all XZ and YZ views are single plane sections. ***p < 0.001.

Tissue samples from tumor (T1), the premalignant condition known as Barrett's Dysplasia (B2) and matched normal proximal mucosa (N1 and N2) were harvested from two freshly resected esophagi. The nature of tissues was verified by histo‐pathological examination of immediately adjacent tissue. Both patients exhibited a higher CTSB expression in the diseased tissue (T1/B2) compared to the normal tissue (N1/N2) as quantified by western blot (Figure 3b). Applying the nanoneedle sensor to the tissues for 15 min allowed for the discrimination of normal samples from diseased ones; the latter displaying a higher cytosolic fluorescence (Figure 3c). The mapping definition within the tissue approached that of a single cell and in many instances allowed for the distinction of cell boundaries and consequently the quantification of the individual cell fluorescence within the tissue (Figure 3c).

Esophageal mucosa from a third patient offered an opportunity to map areas of high and low CTSB activity in the same portion of tissue excised from the margin region of the tumor. Tissue from this patient displayed an increased CTSB expression in the tumor region compared to the healthy region (Figure 3d). Applying the sensor across the portion of tissue including the visible tumor margin (Figure 3e, Figure S6, Supporting Information) allowed for the identification of three key regions (Figure 3e–j). Within the tumor, we detected a CTSB +ve area, displaying uniformly elevated cytosolic fluorescence (T, Figure 3f‐h). A second area within the visually healthy region of the sample was identified as CTSB −ve, showing uniformly low cytosolic fluorescence (N, Figure 3f, Figure S7, Supporting Information). The third region, located in between the CTSB +ve and CTSB −ve regions, presented interspersed CTSB +ve (B+) and CTSB −ve (B−) areas in close proximity to one another (Figure 3f,i,j). These interspersed CTSB areas did not appear to be artifacts due to nanoneedles interfacing, as they differed from the demarcations occurring at the edge of the chip, did not follow straight lines, and were not associated with sharp slopes on the tissue surface at the demarcation (Figure 3a,i,j). The interspersed CTSB +ve and −ve areas will require further analysis of a larger cohort in correlation with histopathology in order to determine the value of biomarker mapping in detecting tumor margin.

This study developed a biosensor based on biodegradable nanoneedles capable of mapping intracellular CTSB activity in human tissue. The mapping achieved single cell resolution in culture and provided a robust strategy to rapidly distinguish cell phenotype in mixed culture. Resolution approached that of a single cell within tissue and allowed the observation of sharp demarcations between adjacent areas with different CTSB activity. These findings suggest that nanoneedles can be developed into a platform in cancer diagnostics that aim at a rapid stratification to identify critical areas for an in‐depth analysis and targeted biopsies.

The choice of the esophagus for this proof‐of‐principle study illustrates a potential deployment strategy of CTSB nanoneedles for the screening of malignant changes in patients with Barrett's dysplasia. These patients carry a 30‐ to 60‐fold increased risk of developing esophageal adenocarcinoma.^26^ Barrett's patients undergo regular endoscopic surveillance and multiple level biopsies to determine disease progression. A nanoneedle sensor applied to the esophageal epithelium during endoscopy could potentially guide the selection of the biopsy sites in order to avoid sampling errors and misdiagnosis. Furthermore, esophageal cancer may be missed at endoscopy in up to 7.8% of patients who are subsequently diagnosed with cancer.^27^ The nanoneedle sensor may thus potentially help endoscopists to avoid missing subtle early cancers.

Combined with our recent assessment of the nanoneedle interface^8^ and the development of a nanoneedle device for genetic engineering in vivo,^3^ the current study defines nanoneedles as a unified platform capable of highly localized delivery of a wide array of bioactive agents and mapping of the inner workings of cells without altering the target tissue. These findings demonstrate the versatility of nanoneedles to mediate the interaction within the intracellular space in complex organisms. This novel technology holds great promise for highly localized in vivo bioengineering at the bedside.

## Experimental Section

*Assembly of Nanoneedles Sensor*: Nanoneedles were fabricated as previously reported.^3^, ^4^ The nanoneedles were first oxidized by O_2_ plasma for 10 min (0.4 mTorr, 75W, Diener plasma asher) and then treated with 2% v/v 3‐aminopropyltriethoxysilane in ethanol for 2 h, and thoroughly washed with ethanol. The samples were incubated for 2 h with 10 × 10^−6^ m 1:4 SMPEG‐12:MSPEG12 (thermo pierce, USA) in PBS at RT, washed, incubated for 1.5 h with 0.01 mg mL^−1^ of CFK‐lys‐TAMRA peptide (Lifetein, USA) in PBS, washed three times for 15 min in PBS, and once in DI water. The sensors were dried under N_2_ stream and stored at 4 °C for up to a week.

*Nanoneedles Sensing in Culture*: Culture medium was removed from the culture in a two‐well chamberslide, leaving a thin layer over the cells. The nanoneedle chip was placed over the culture and centrifuged at 150 rcf for 1 min. Fresh medium was added and the culture was incubated for the desired time. LSC imaging was performed on the chip following the fixation of cells with 4% w/v PFA for 15 min. Flow cytometry was performed on cells after trypsinisation from the chip and fixation in 1% w/v PFA for 15 min.

*Nanoneedles Sensing in Tissue*: The study employed human tissue samples stored by the Imperial College Healthcare Tissue Bank. The study was approved by the institutional review board at the Imperial College National Healthcare Service Trust (UK Research Ethics Committee reference 04/Q0403/119, project reference R12063). The tissue was thawed at room temperature and rapidly interfaced with nanoneedles applying manual pressure. The interfaced tissue sample was incubated for 15 min at 37 °C in a tissue culture incubator, when the chip was removed. The tissue was immediately stained with DAPI for 10 min and imaged by LSC following immersion into Hanks balanced salt solution.

The data presented in Figure 1c,d originate from the same set of confocal and SEM experiments previously shown in ref.^3^.

Detailed methods are available in the Supporting Information.

## Supporting information

As a service to our authors and readers, this journal provides supporting information supplied by the authors. Such materials are peer reviewed and may be re‐organized for online delivery, but are not copy‐edited or typeset. Technical support issues arising from supporting information (other than missing files) should be addressed to the authors.

Supplementary
