# Supplementary material for: Mapping Local Cytosolic Enzymatic Activity in Human Esophageal Mucosa with Porous Silicon Nanoneedles
Source: Adv Mater. 2015 Jul 21;27(35):5147–52. doi: 10.1002/adma.201501304 (PMC4858817; doi:10.1002/adma.201501304)
Supplement: Supplementary file 1 — Supplementary [file ADMA-27-5147-s001.pdf]

Copyright WILEY-VCH Verlag GmbH & Co. KGaA, 69469 Weinheim, Germany, 2015.

ADVANCED MATERIALS

Supporting Information

for *Adv. Mater.,* DOI: 10.1002/adma.201501304

# Mapping Local Cytosolic Enzymatic Activity in Human Esophageal Mucosa with Porous Silicon Nanoneedles

*Ciro Chiappini,* Paola Campagnolo, Carina S. Almeida, Nima Abbassi-Ghadi, Lesley W. Chow, George B. Hanna, and Molly M. Stevens**

Copyright WILEY-VCH Verlag GmbH & Co. KGaA, 69469 Weinheim, Germany, 2013.

# Supporting Information

## Mapping Local Cytosolic Enzymatic Activity in Human Esophageal Mucosa with Porous Silicon Nanoneedles

*Ciro Chiappini*, Paola Campagnolo, Carina S. Almeida, Nima Abbassi-Ghadi, Lesley W. Chow, George B. Hanna, Molly M. Stevens*.*

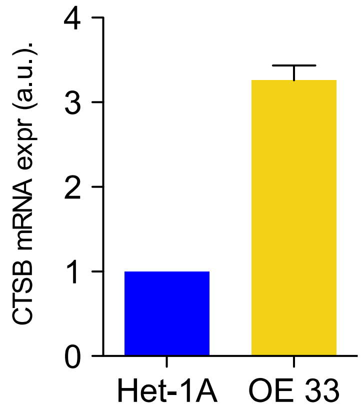


**Figure S1.** Relative expression of CTSB mRNA for HET-1A and OE33 cells as assayed by qPCR.

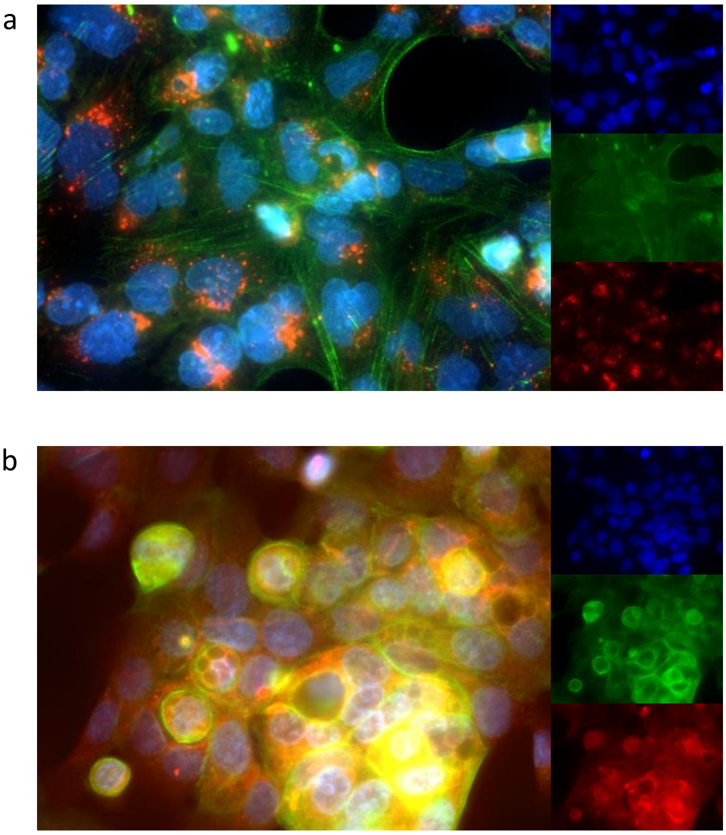


**Figure S2** CTSB localization by immunofluorescence. Epifluorescence micrographs of (a) Het-1A and (b) OE33 cells in culture. Blue, nuclei; green, actin; red, CTSB.

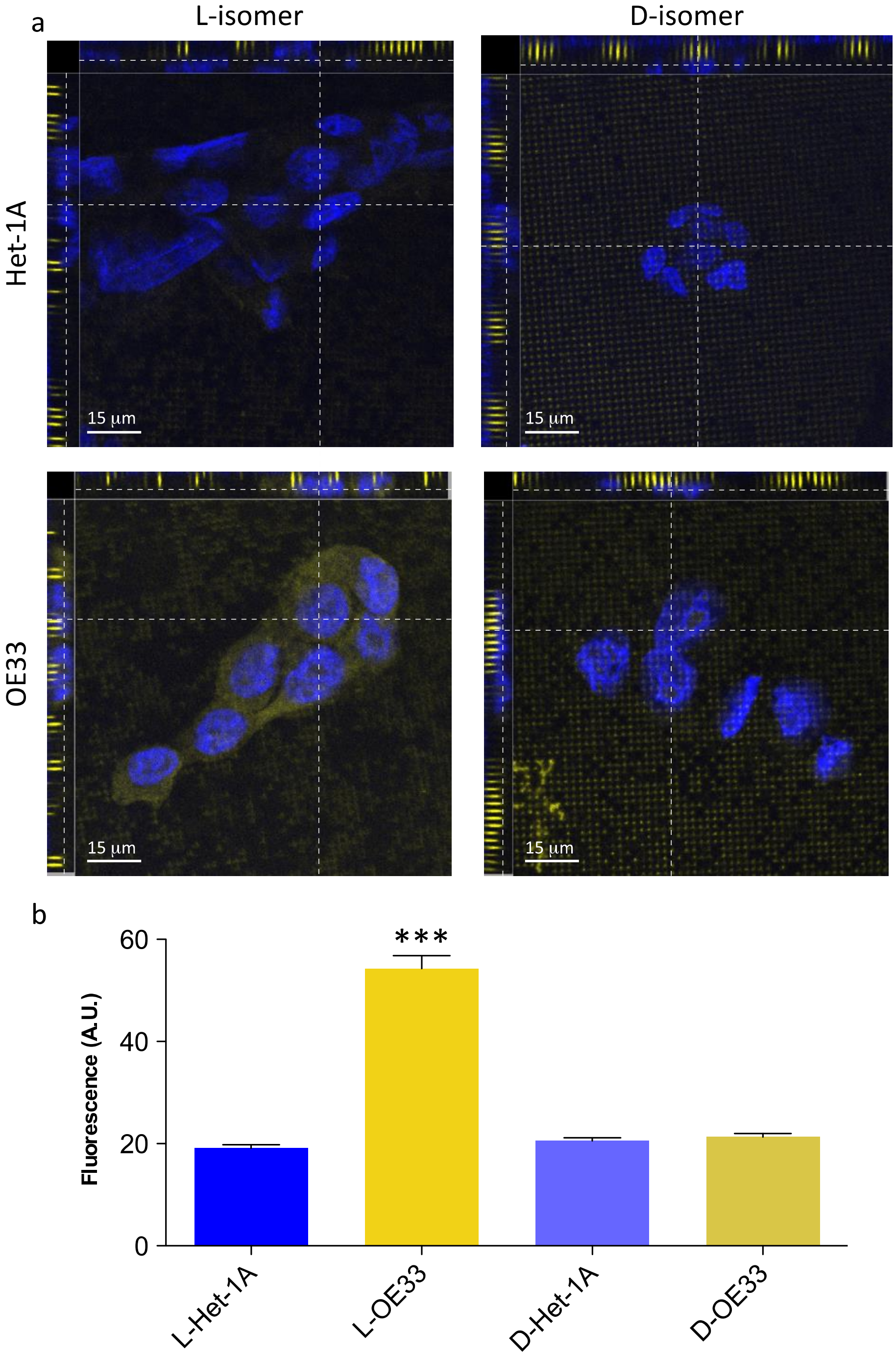


**Figure S3** Cytosolic fluorescence originates from proteolytic cleavage of the L-isomer sensing element. (a) Scanning confocal micrographs of Het-1A (top) and OE33 (bottom) cells interfaced with nanoneedles sensors equipped with a L-isomer CFKK-TMR peptide (left) or a D-isomer CFKK-TMR peptide. (b) Quantification of cytosolic fluorescence. Data is gathered from at least 20 cells and five separate confocal micrographs for each sample. $p< 0.01$ *w.r.t.* all other samples.

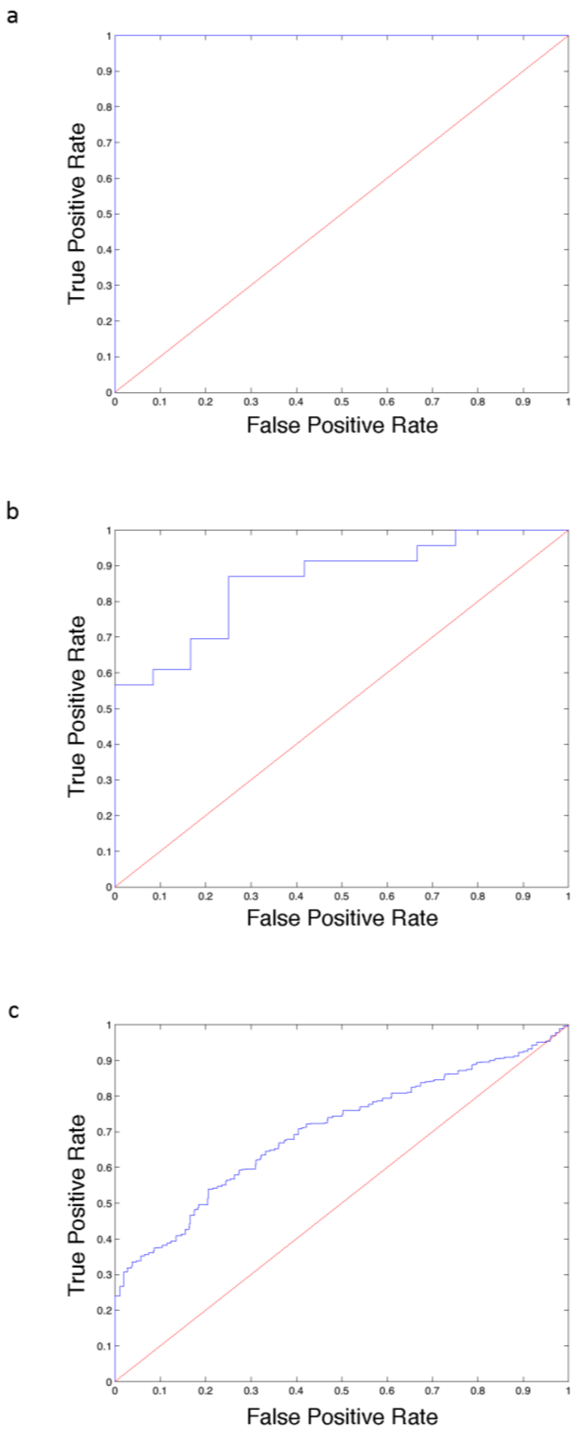


**Figure S4.** Receiver operator characteristics for CTSB sensing by nanoneedles for (a) confocal microscopy quantification on single cultures of OE33 and Het-1A cells at 15 minutes interfacing, (b) confocal microscopy quantification on co-cultures of OE33 and Het-1A at 15 minutes interfacing, (c) flow cytometry quantification on co-cultures of OE33 and Het-1A at 15 minutes interfacing. ROC for the classifier of interest is shown in blue, red line represents 0.5 AUC. Panel (a) with an AUC of 1 is representative of all interfacing times for single cultures.

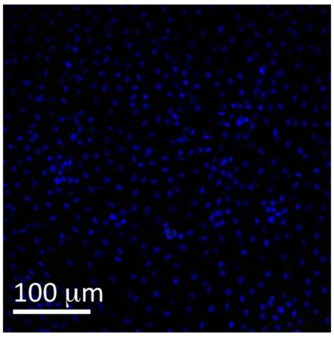


**Figure S5** LSC of esophageal tissue following 15 minutes of interfacing with a flat silicon chip equipped with CTSB sensing element. Blue, nuclei; yellow, CTSB fluorescent substrate.

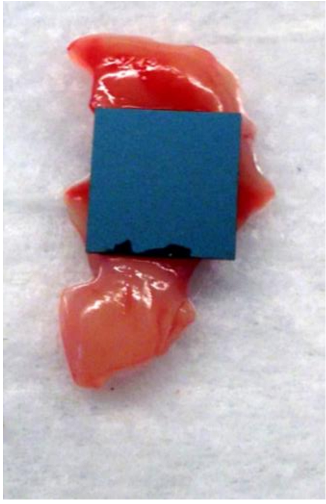

**Figure S6** Photograph of the nanoneedle sensor while interfaced with the tissue margin sample shown in Figure 3e.

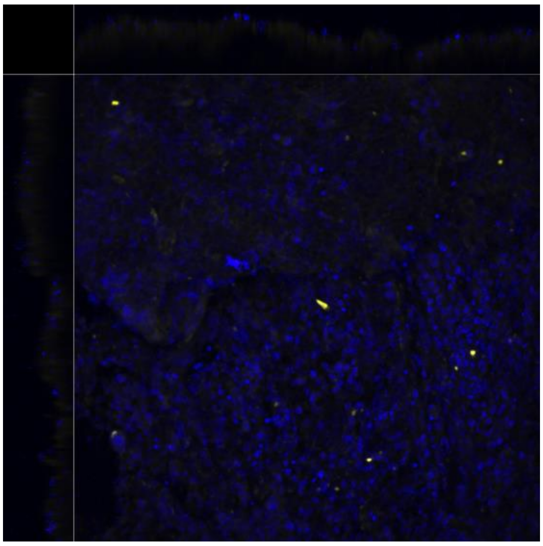

**Figure S7** Normal region of the margin tissue sample depicted in Figure 3e following 15 minutes interfacing with nanoneedles. Blue, nuclei; yellow, CTSB fluorescent substrate.

**Supplementary Methods**

*Sensor testing in vitro*: The nanoneedle sensor was incubated with 100 µl of either Cathepsin B at the desired unit concentration or controls in pH 6.0 phosphate buffer at 37 °C in humid atmosphere for the desired time. A total of 90 µl of the supernatant was collected and transferred to a 96 well plate. The fluorescence was measured with a plate reader (PerkinElmer, USA) equipped with gratings to excite the samples at 540 nm and collect emission at 590 nm. The linear regressions were calculated with MATLAB (Mathworks Inc.) and compared by ANCOVA.

*OE33 cell culture conditions*: OE33 cells were cultured with RPMI 1640 medium supplemented with L-glutamine, 10% v/v fetal calf serum and antibiotic/antimicotic. Cells were passaged when reaching greater than 75% confluency.

*Het-1A cell culture conditions*: Het-1A cells were cultured with DMEM medium supplemented with glutamax, 10% v/v fetal calf serum and penicillin/streptomycin. Cells were passaged when reaching greater than 75% confluency.

*Co-culture conditions*: Co-cultures were obtained mixing 60% OE33 cells with 40% HET-1A cells in RPMI 1640 medium supplemented with l-glutamine, 10% v/v fetal calf serum and penicillin/streptomycin. The OE33 cells were fluorescently labeled immediately prior to mixing to insure recognition in co-culture either with celltracker green (Invitrogen, USA) for flow cytometry or with cellvue NIR 780 nm (eBioscience, Ltd., UK) for confocal microscopy. Mixed cultures were used exclusively at endpoint experiments and never passaged.

*Homogenous Cathepsin B activity assay*: Cathepsin B buffer was prepared as 0.1 M phosphate pH 6.2 with 4mM Cys, 5 mM EDTA, and 1% v/v triton-X100. Cells cultured in 12 well plates were lysed with 200 µl of Cathepsin B buffer at 4 °C, scraped from the well and maintained refrigerated while vortexing them every 10 minutes for 30 minutes, to promote cell disruption. The cell debris was removed by centrifugation at 1000 x g for 5 minutes. The

supernatant was collected and employed for the Cathepsin B activity assay. In a 96 well plate, 100 µl of cell lysate was added to 2 µl of z-rr-amc fluorogenic Cathepsin B substrate, and the reaction was monitored in a plate reader (PerkinElmer) for 5 min at 40 °C with excitation at 384 nm and emission at 440 nm. A separate well from the same plate was trypsinised and employed to count cells for normalisations. Experiments were run in triplicate. Statistical analysis was performed by t-test in GraphPad Prism.

*Cathepsin B immunofluorescence*: Cells were grown in an 8-well chamber slide until reaching greater than 70% confluence. Cells were washed three times with PBS and fixed in methanol at -20 °C for 10 minutes. Cells were permeabilized and blocked with 1% v/v bovine serum albumin, 10% v/v goat serum, 0.3M glycine, and 0.1% v/v tween in PBS for 1 hour at room temperature. The cells were incubated with anti-Cathepsin B antibody raised in rabbit (ab92955, AbCam) in blocking buffer overnight at 4 °C, followed by washing five times with PBS. Secondary antibody staining was obtained with AlexaFluor 594 anti-rabbit IgG for 2 hours in PBS, followed by three washes in PBS. Actin was stained with AlexaFluor 488 conjugated phalloidin for 15 minutes. The samples were mounted on a coverslip with ProlongGold with DAPI overnight, and imaged by epifluorescence the following day.

*Confocal microscopy of cells*: Cells were interfaced with nanoneedles as described in the experimental section of the main text. The cells were then fixed in 4% w/v paraformaldehyde and stained with DAPI for 3 minutes. The samples were mounted on a coverslip with ProlongGold overnight and imaged by confocal microscopy (Leica SP5, Leica GMBH, Germany) the following day. Cathepsin B signal was acquired upon excitation with 543 nm HeNe laser line and acquisition in the 560 nm to 630 nm range at 32% laser power and constant photomultiplier gain and offset for all samples considered in a single plane above the tip of the nanoneedles and passing through the cell nucleus. Between 8 and 42 cells from at least 5 fields of view were acquired for each sample at each time. The total fluorescence originating from each cell was normalized by its area and employed as the classifier. The data

was analyzed by two-way ANOVA with prism. Graphs are reported as mean with standard error of the mean. To estimate the validity of the classifier, the classifier for each of the cells analyzed in each sample together with the true value of cell type (OE33 or Het-1A) was subdivided into two train and test groups. The train group was employed to identify the parameters of the logistic regression, which was then applied to the test group. The ROC graphs and AUC reported were calculated with Matlab and refer to the test group in all instances.

*Flow cytometry of cells*: Cells were interfaced with nanoneedles for 15 minutes as described in the experimental section of the main text. The cells were trypsinized off the nanoneedles and fixed in 1 ml of 1% w/v paraformaldehyde in PBS. The samples were immediately analyzed by flow cytometry (Fortessa, BD, UK), employing the same cytometry settings. Cytosolic fluorescence originated from CTSB cleavage of the substrate peptide was collected with laser excitation at 561 nm and emission at 582 nm with a 15 nm window, while cell staining to identify OE33 cells was collected at 488 nm and emission at 530 nm with a 30 nm window. The CTSB fluorescence of all cell events in each sample together with the true value of cell type from the OE33 staining was subdivided into two train and test groups. The train group was employed to identify the parameters of the logistic regression, which was then applied to the test group. The ROC graphs and AUC reported were calculated with Matlab and refer to the test group in all instances.

*RNA and protein extraction:* Total RNA was extracted from cells using RNeasy Mini Kit (Qiagen) following manufacturer's instructions. To extract the proteins, cell pellets were resuspended in RIPA buffer and sonicated. Tissues were snap frozen in liquid nitrogen, grounded and homogenized, and then resuspended in Trizol (Life technologies). Both RNA and proteins were obtained from each tissue sample. After phase separation, RNA was

extracted from the aqueous phase and proteins were obtained from the organic phase, following sequential washing steps and final resuspension.

Protein quantification was performed by DC protein assay (Biorad) and RNA was quantified using Nanodrop spectrophotometer (Thermo) and employed for downstream investigations.

*Reverse transcription and gene expression analysis*: For each sample, 500 ng of RNA was reverse transcribed using QuantiTect (Qiagen) reverse transcription kit according to manufacturer's instructions. Real time PCR was performed in a Rotor-Gene Q (Qiagen) using the QuantiTect SYBR Green PCR mix (2X, Qiagen) and the following primer sets: for Cathepsin B Forward-CCAGGGAGCAAGACAGAGAC and Reverse-GAGACTGGCGTTCTCCAAAG and for GAPDH Forward-TGGTATCGTGGAAGGACTCATGA and Reverse-ATGCCAGTGAGCTTCCCGTTCAG.

*Protein Analysis*: Equal amount of proteins were loaded on pre-cast Mini-PROTEAN (Bio-Rad) gels and run at 100 mV for 90 minutes. Separated proteins were transferred on Protran membrane and incubated with specific antibodies overnight at 4 °C, after blocking with 5% v/v milk. Cathepsin B antibody was purchased from Santa Cruz (sc13985, 1:200) and β-actin (sc47778, 1:500). Following secondary antibody incubation with IRDye antibodies (LI-COR Biosciences), images were acquired at the Odyssey infrared scanner (LI-COR Biosciences).

*Interfacing flat silicon chips with tissue*: a pristine <100> 0.01 Ω-cm p-type silicon wafer was diced into 8x8mm dies. Cy3 NHS ester was functionalized with the same protocol described for obtaining nanoneedles sensors. The chip was applied to a portion of tissue applying pressure by hand. The applied chip was incubated for 15 minutes at 37 °C in a tissue culture incubator, the chip was removed, the tissue was DAPI stained for 10 minutes and placed in Hanks balanced saline solution.

*Confocal microscopy of tissues*: Immediately following treatment the tissue samples were stained for DAPI for 10 minutes and moved to hanks balanced saline solution in 6-well plates

and imaged by confocal microscopy (Leica SP2 equipped with 25x, 0.9NA water dipping objective Leica GMBH, Germany). Micrographs were collected with a 3 μm step over a z-stack that included the entire surface of the tissue within the field of view. Magnification, laser power, acquisition window and photomultiplier gain were maintained constant for all samples acquired within the same session, which included all matched samples: flat chip and nanoneedles comparison in one session, N1, N2, T1, B2 from patients 1 and 2 in one session, N, B, T from patient 3 in one session. The z-stack was merged by maximum projection and the resulting images were analyzed for cytosolic fluorescence of cells. For patients 1 and 2, a total of at least 30 cells from at least 3 fields of view were acquired for each sample. The total fluorescence originating from each cell was normalized by its area and employed as the classifier. The data was analyzed by ANOVA with GraphPad Prism. Graphs are reported as mean with standard error of the mean. For patient 3, the average fluorescence from each image collected in the N and T region was calculated and used for the average reported. The images collected in the B region were manually segmented into two areas of high (B+) and low (B–) fluorescence. The average from each area was used to estimate the average fluorescence for B+ and B–, respectively. The samples were analysed by ANOVA in GraphPad Prism. Data is reported as mean with standard error of the mean (SEM).

*Comparison of D- and L-isomer sensors*: The D-isomer of CFKK-TMR was synthesized in-house according to standard solid phase peptide synthesis protocols, using D-analogs of the amino acids (Anaspec, USA) and Tetramethylrhodamine NHS ester (Sigma-Aldrich). The peptide was conjugated to the nanoneedles according to the same protocol employed for the L-isomer described above. Both L- and D-isomer sensors were interfaced with OE33 and Het-1A cells for 15 minutes according to the interfacing protocol described. The sensors were removed, fixed in 4% w/v paraformaldehyde in PBS for 15 minutes, and counterstained with DAPI for 5 minutes. The samples were mounted on coverslip with prolong gold (Life

Technologies) and stored at 4 °C overnight. On the following day the samples were imaged by confocal microscopy. Five separate z-stacks including the nanoneedles and the entirety of the cells were imaged for each sample. The DAPI and TMR channels were acquired sequentially to avoid cross-talk. All stacks were acquired with constant magnification, laser intensities, detector gain and offset on all channels. The data was quantified for all cells in all images from a single Z plane above the needles as described in the *confocal microscopy of cells* section.
